# Genetic, biochemical, and structural characterization of a novel KLUS subgroup of CTX-M-type extended-spectrum β-lactamases reveals an evolutionarily distinct lineage with unique functional properties

**DOI:** 10.3389/fmicb.2026.1997337

**Published:** 2026-10-30

**Authors:** Zhenzhou Huang, Jun Li, Xiao Liu, Lingyi Zeng, Zhibei Zheng, Hua Yu, Duochun Wang, Keyi Yu, Junfang Chen

**Affiliations:** 1 Hangzhou Center for Disease Control and Prevention (Hangzhou Health Supervision Institution), Hangzhou, Zhejiang, China; 2 Zhejiang Key Laboratory of Multi-Omics in Infection and Immunity, Hangzhou, Zhejiang, China; 3 National Key Laboratory of Intelligent Tracking and Forecasting for Infectious Diseases, National Institute for Communicable Disease Control and Prevention, Chinese Center for Disease Control and Prevention and Chinese Academy of Preventive Medicine, Beijing, China; 4 Department of Microbiology, Zhejiang Provincial Center for Disease Control and Prevention, Hangzhou, Zhejiang, China; 5 NHC Specialty Laboratory of Food Safety Risk Assessment and Standard Development, Zhejiang Provincial Center for Disease Control and Prevention, Hangzhou, China; 6 Zhejiang Key Laboratory of Public Health Detection and Pathogenesis Research, Zhejiang Provincial Center for Disease Control and Prevention, Hangzhou, China

**Keywords:** antimicrobial resistance, CTX-M-type β-lactamases, enzyme kinetics, KLUS, molecular evolution

## Abstract

Extended-spectrum β-lactamases (ESBLs), particularly the CTX-M family, have progressively undermined the clinical utility of β-lactam antibiotics. Herein, we identified and characterized a novel CTX-M subgroup designated KLUS, and investigated its taxonomic position and functional properties. Phylogenetic analysis combined with genetic distance calculations supported KLUS as a distinct lineage, with inter-subgroup distances significantly exceeding intra-subgroup distances and distinct clustering (bootstrap support >95%), consistent with the previously established family-wide CTX-M classification. Recombinant plasmids harboring each of the four *bla*_KLUS_ alleles were constructed in *E. coli* BL21 (DE3), with optimal expression achieved using 1.0 mM IPTG at 30 °C for 5 h. Minimum inhibitory concentration (MIC) determinations revealed that KLUS-1 through KLUS-3 conferred high-level resistance to ampicillin and ceftriaxone, moderate resistance to cefotaxime, and minimal resistance to ceftazidime (MIC < 0.125 μg/mL). KLUS-4 exhibited enhanced ceftazidime resistance (MIC = 8 μg/mL) and a reduced inhibitor response at the phenotypic level (a 16-fold reduction of the cefotaxime MIC with clavulanic acid, in contrast to KLUS-1, where clavulanic acid reduced the MIC to <0.015 μg/mL). β-lactamase inhibitors reduced cefotaxime MICs far exceeding 16-fold in KLUS-1 through KLUS-3, consistent with ESBL phenotypes. Steady-state kinetic analyses demonstrated subtype-dependent catalytic efficiencies that were highest for ampicillin and penicillin G in KLUS-2/3/4 and for ceftriaxone in KLUS-1, broadly consistent with MIC results. The substantially lower catalytic efficiency for ceftazidime may explain its reduced resistance phenotype. Structural modeling revealed that KLUS enzymes comprise 11 α-helices and 5 β-sheets, with a conserved catalytic cavity and SDN loop. Twenty-three unique amino acid substitutions distinguish KLUS from CTX-M-2. V77I (pre-protein positions 106) is located proximal to the Ω-loop region, and any functional contribution of this conservative substitution remains speculative. These findings expand the known diversity of CTX-M β-lactamases; the clinical significance of KLUS requires further investigation in clinical isolates.

## Introduction

β-lactam antibiotics constitute a large class of antimicrobial agents defined by the presence of a β-lactam ring in their chemical structure (Cho et al., 2014). This class includes clinically predominant penicillins, cephalosporins, and newly developed agents such as cephamycins, thienamycins, monobactams, and other atypical β-lactam antibiotics (Qin et al., 2014). They offer distinct advantages, including potent bactericidal activity, low toxicity, broad clinical indications, and favorable therapeutic efficacy. These antibiotics exert their antimicrobial effects by inhibiting penicillin-binding proteins (PBPs), thereby disrupting peptidoglycan synthesis in the bacterial cell wall, leading to cell wall defects, cellular swelling, and eventual lysis (Zapun et al., 2008). A wide range of Gram-negative bacterial genera exhibit either intrinsic or acquired resistance to these antibiotics. Selective pressure resulting from the clinical use and overuse of antibiotics has driven the selection and emergence of novel β-lactamase variants. The escalating antimicrobial resistance, particularly that mediated by class A ESBLs, has become a major global public health challenge (Lee et al., 2012). *Enterobacteriaceae* strains producing ESBLs are resistant to penicillins, aztreonam, and first-, second-, and third-generation cephalosporins, but lack the ability to hydrolyze cephamycins or carbapenems (Doi et al., 2017). Class A ESBLs primarily encompass the TEM, SHV, CTX-M, VEB and GES enzyme families (Bush, 2018). Among these, the CTX-M family has undergone remarkable expansion in variant subtypes in recent years, giving rise to the so-called ‘CTX-M pandemic’ worldwide (Cantón et al., 2012). The CTX-M family constitutes a complex and heterogeneous group of enzymes.

CTX-M-type ESBLs serve as a paradigm for the evolution of drug resistance mechanisms. CTX-M-type β-lactamases were first detected in Europe and South America (Bauernfeind et al., 1992; Bauernfeind et al., 1990). They were successively identified in South America (Bauernfeind et al., 1996), Europe (De Champs et al., 2000; Gniadkowski et al., 1998; Pałucha et al., 1999), and Asia (Yan et al., 2000)between 1992 and 1999. Since the 21st century, CTX-M-type ESBLs have shown a pattern of accelerated evolution and abnormal dissemination, with hotspots concentrated in Central Europe, South America, and Japan. This phenomenon is not only related to region-specific selective driving forces but may also be associated with their abundance in organisms of specific clones or sources, reflecting the geographical distribution specificity of different enzymes. Driven by antibiotic selection pressure and gene recombination (Cantón et al., 2012), CTX-M-type extended-spectrum β-lactamases exhibit significant type diversity, with more than 280 types identified so far. Vertical gene transmission enables the inheritance and continuation of the parental antibiotic resistance gene pool in offspring. Relevant studies have shown that CTX-M-type ESBLs originate from the chromosomes of *Kluyvera* spp. (Rodríguez et al., 2021; Yu et al., 2023). The gene homologs of the six subgroups have strong type associations with different species of *Kluyvera*. The chromosome of *K. cryocrescens* carries genes encoding the CTX-M-1 group and KLUC group; genes encoding the CTX-M-1 group and CTX-M-2 group are mainly detected on the chromosome of *K. ascorbata*; and the chromosome of *K. georgiana* carries genes encoding the CTX-M-8 group, CTX-M-9 group, and CTX-M-25 group (Decousser et al., 2001; Humeniuk et al., 2002; Olson et al., 2005; Poirel et al., 2002; Rodríguez et al., 2010). As the source of resistance gene dissemination, the ancestor bacteria’s ability to harbor specific resistance genes, including the quantity and types of harbored genes, plays an important role in studying the migration and transformation of resistance genes.

In previous studies, we defined CTX-M-type ESBLs into 10 subgroups based on 20 amino acid site variants, and the results of this subgroup division were consistent with phylogenetic clustering (Yu et al., 2023). That family-wide analysis, which encompassed all mature CTX-M sequences available in the BLDB database, established a 10-subgroup classification scheme (intra-subgroup amino-acid identity >93.13%, i.e., ≤20 amino-acid substitutions) validated against the phylogenetic clustering of the complete family (Yu et al., 2023). It was within this framework that KLUS was first proposed as an independent subgroup. Concurrently, through the application of phylogenomics and antibiotic resistance gene screening, we characterized numerous new *bla*_CTX-M_ genotypes and identified a novel CTX-M subgroup, KLUS, which had not been previously recognized. To systematically unravel the biological significance and clinical implications of the KLUS subgroup, our research clarified its taxonomic status through phylogenetic tree topological structure analysis and genetic distance calculation, comparing its evolutionary relationship with the CTX-M-2 subgroup to confirm its independence as a new subgroup, extending the established family-wide classification with detailed functional characterization. Then, we constructed recombinant plasmids containing KLUS subgroup genes, evaluated the enzymatic activity of the KLUS subgroup using nitrocefin hydrolysis assay and steady-state kinetics analysis, and clarified its substrate preference and catalytic efficiency, so as to explain the molecular mechanism of its drug resistance phenotype. Our investigations are poised to deepen our comprehension of the evolutionary diversification mechanisms of CTX-M-type ESBLs, while providing critical theoretical foundations for the rational clinical application of β-lactam antibiotics and the development of novel β-lactamase inhibitors, thereby addressing the escalating challenge of antimicrobial resistance mediated by these enzymes.

## Materials and methods

### Bacterial strains and vectors

*K. sichuanensis* 090646, which harbors *bla*_KLUS-1_ (chromosomally encoded) and *bla*_CTX-M-220_ (plasmid-borne; a CTX-M-2 group member), as well as *bla*_NDM-1_, was isolated from a hospital sink in Chengdu, Sichuan Province, China. These two *bla* genes were distinct alleles belonging to different CTX-M subgroups. This strain exhibited a strong multidrug resistance phenotype, showing resistance to ampicillin, azithromycin, aztreonam, cefotaxime, imipenem, and streptomycin, among other antimicrobial agents. *K. excreta* CHPC 1.251 and *K. sichuanensis* CHPC 1.254 were isolated from fecal samples, while *K. chilikensis* CHPC 1.2972 was recovered from egg surface swabs. The expression vector pET-28a(+) was purchased from Novagen. *Escherichia coli* DH5α and *E. coli* BL21 (DE3) were preserved in our laboratory. All CTX-M sequences were retrieved from the BLDB database.^1^ And the detailed information of strains have been listed in Supplementary Table S1.

### Phylogenetic analysis and genetic distance calculation

Amino-acid sequences of CTX-M-type β-lactamases were retrieved from the BLDB database (footnote 1). A maximum-likelihood phylogenetic tree was reconstructed in MEGA X with 1,000 bootstrap replicates. Pairwise evolutionary distances within and between subgroups were calculated in MEGA X. Statistical comparisons of intra- versus inter-subgroup distances were performed using the Mann–Whitney U test in GraphPad Prism 9, with significance set at *p* < 0.05. The genetic environment of the *bla*_KLUS_ alleles was characterized from the source genome assemblies as described previously (Yu et al., 2023).

### Construction and validation of recombinant plasmids

In line with the sequence characteristics of the expression vector pET-28a(+), a BamHI restriction site was incorporated into the 5′ end of the upstream primer specific for the KLUS gene, while a HindIII restriction site was introduced into the 5′ end of the corresponding downstream primer. PCR amplification was performed using the primer sequences detailed in Supplementary Table S2. The ligation product, comprising the plasmid vector and the target gene, was transformed into competent *E. coli* DH5α cells. Amplicons were verified by Sanger sequencing. DNA sequencing of PCR-positive clones confirmed 100% sequence identity to the original KLUS gene sequences. Furthermore, the fragment sizes yielded by double restriction enzyme digestion (BamHI/HindIII) were consistent with the theoretically expected lengths.

### Protein expression and optimization of expression conditions

*In silico* analysis performed using DNAstar software demonstrated that genes of the KLUS subgroup encode proteins with an average molecular weight (MW) of 31.50 kDa, with the molecular weights of individual subtypes as follows: KLUS-1 (32.29 kDa), KLUS-2 (31.29 kDa), KLUS-3 (31.19 kDa), and KLUS-4 (31.23 kDa). The theoretical isoelectric point (pI) of all KLUS subgroup proteins was calculated as 7.6. After *in silico* prediction and removal of the signal peptide, the mature proteins exhibited molecular weights of 29.17 kDa, 28.2 kDa, 28.1 kDa, and 28.14 kDa for KLUS-1 through KLUS-4, respectively.

For protein expression, the recombinant plasmid pET-28a-KLUS was transformed into *E. coli* BL21 (DE3). Positive clones were initially induced with 1 mM IPTG at 37 °C for 4 h. Subsequent SDS-PAGE analysis revealed specific bands at the anticipated positions, consistent with the predicted molecular weights of the recombinant fusion proteins. To optimize the expression conditions, a series of induction experiments were carried out: induction with gradient concentrations of IPTG (0.5, 1.0, and 2.0 mmol/L) at 37 °C, and with 1.0 mmol/L IPTG at different temperatures (16 °C for 18 h, 30 °C for 5 h, and 37 °C for 4 h). SDS-PAGE was employed to assess the protein expression levels under each condition, thereby facilitating the determination of the optimal IPTG concentration and induction temperature. Optimal expression was achieved with 1.0 mM IPTG at 30 °C for 5 h. After confirming the optimal expression conditions, scale-up cultivation of the target protein was performed.

The target protein was purified and concentrated using the Novagen His•Bind® Purification Kit following the manufacturer’s recommended protocols. The concentration of the purified target protein was quantified via the BCA method using the Thermo Scientific™ Micro BCA™ Protein Assay Kit. Total β-lactamase activity (nitrocefin hydrolysis; Δε482 = 15,000 M^−1^ cm^−1^) and total protein were measured at each purification step to derive total activity, specific activity, fold-purification, and recovery (Supplementary Table S3).

### Determination of minimum inhibitory concentration (MIC) for recombinant strains

The drug resistance phenotypes of the recombinant strains were determined using the micro-broth dilution method. Antimicrobial agents were subjected to twofold serial dilution in Mueller-Hinton (MH) broth. Bacterial cultures were adjusted to a concentration of 10^5^ colony-forming units CFU/mL in IPTG-supplemented MH broth (1 mmol/L), and 100 μL of this bacterial suspension was added to each well of the microtiter plate, resulting in a final total volume of 200 μL per well.

Concurrently, a control group was established using bacterial cultures diluted to 10^5^ CFU/mL in MH broth to exclude the potential impact of IPTG on bacterial growth. *E. coli* ATCC 25922 was used as the standard quality control strain to ensure the validity of the assay. Additional control groups were included to ensure assay rigor: blank MH broth (negative control), bacterial cultures without antimicrobial agents (growth control), and antimicrobial agent solutions without bacterial inoculum (drug solution control). β-Lactamase inhibitors (clavulanic acid, tazobactam, and sulbactam) were used at a fixed concentration of 4 μg/mL, following the CLSI-standard format for inhibitor-combination susceptibility testing. All MIC determinations were performed in three independent biological replicates, and modal values are reported; replicate ranges never exceeded one twofold dilution.

### β-lactamase activity assay

β-lactamases catalyze the hydrolysis of the amide bond linking the carbonyl carbon and nitrogen within the β-lactam ring of nitrocefin, a chromogenic substrate. β-Lactamase activity was confirmed using nitrocefin hydrolysis. A stock solution of nitrocefin (10 mg/mL) was prepared in dimethyl sulfoxide (DMSO) and subsequently diluted to a working concentration of 1 mg/mL using phosphate-buffered saline (PBS). Following induction of the recombinant strains, 1 mg/mL nitrocefin working solution was added to the bacterial cultures. Development of a red color confirmed active β-lactamase production. The absorbance was measured at a wavelength of 482 nm.

### Steady state kinetics

All spectrometric data were acquired using a Cary 60 UV–Vis spectrophotometer. Substrates for steady-state velocity determination were freshly prepared in 50 mM phosphate buffer. Enzymatic reactions were initiated by mixing the purified enzyme with substrate (final concentrations spanning 100–1,000 μM) in a total reaction volume of 200 μL, yielding a final enzyme concentration of 50 μg/mL. Initial reaction velocities were determined from the linear segment of the reaction progress curve. Experimental data were plotted as initial velocity versus substrate concentration, and the Michaelis constant (*K*_m_) and maximum reaction velocity (Vmax) values were derived via nonlinear regression analysis using the Michaelis–Menten equation. The catalytic constant (*k*_cat_) was calculated by dividing Vmax by the total enzyme concentration, in accordance with established protocols. The molar extinction coefficients and optimal wavelengths (Laraki et al., 1999) for each substrate are detailed in Table 1. All kinetic measurements were performed in three independent experiments with separately purified enzyme preparations. Because active-site titration was not performed, all *k*_cat_ values are reported as apparent *k*_cat_ estimates calculated from total enzyme concentration. For low-turnover substrate–enzyme pairs for which substrate solubility precluded full saturation (final substrate concentrations ≤1,000 μM), *K*_m_ values are reported as apparent, lower-bound estimates.

**Table 1:** The molar extinction coefficient of antibiotic at optimum measured wavelength.

Antibiotic	Molar extinction coefficient
Ceftriaxone	Δε_241 nm_ = 495–545 M^−1^ cm^−1^
Cefotaxime	Δε_264 nm_ = −7,250 M^−1^ cm^−1^
Ceftazidime	Δε_260 nm_ = −9,000 M^−1^ cm^−1^
Nitrocefin	Δε_482 nm_ = 15,000 M^−1^ cm^−1^
Ampicillin	Δε_235 nm_ = −900 M^−1^ cm^−1^
Penicillin G	Δε_235 nm_ = 775 M^−1^ cm^−1^

The nitrocefin molar extinction coefficient of 15,000 M^−1^ cm^−1^ at 482 nm applies to measurements in neutral phosphate buffer.

### Protein structure analysis

Based on the amino acid sequences of genes belonging to the KLUS subgroup, the SWISS-MODEL online platform^2^ was utilized to predict the three-dimensional spatial structures of the encoded proteins. Phylogenetic analysis indicated that the genes within the KLUS subgroup exhibit the highest sequence similarity to those of the CTX-M-2 subgroup. The CTX-M-2 protein sequence, retrieved from the UniProt database was employed as the template to evaluate the feasibility of homology modeling. All Global Model Quality Estimation (GMQE) values were greater than 0.9, demonstrating the generation of high-quality predicted models. Furthermore, PyMOL v2.5.7 software was used for detailed protein structure analysis.

## Results

### Identification and taxonomic position of the KLUS subgroup

Phylogenetic tree topological analysis revealed that CTX-M-type ESBLs could be divided into multiple subgroups, consistent with previous observation (Figure 1A) (Yu et al., 2023). The tree includes representative members of all established CTX-M subgroups. An enlarged rectangular cladogram with strain identifiers and bootstrap values at all nodes is provided in Supplementary Figure S1. Most CTX-M subtypes displayed a convergent evolution pattern, and intra-subgroup variants differed by only single amino acid residues, indicative of evolutionary conservation across subtypes within the same subgroup. KLUS subgroup sequences clustered adjacent to those of the CTX-M-2 subgroup yet formed a distinct independent clade with a notable branch length. Bootstrap support for this branching pattern exceeded 95%, implying a close genetic affinity between these two subgroups alongside substantial evolutionary divergence.

**Figure 1:**
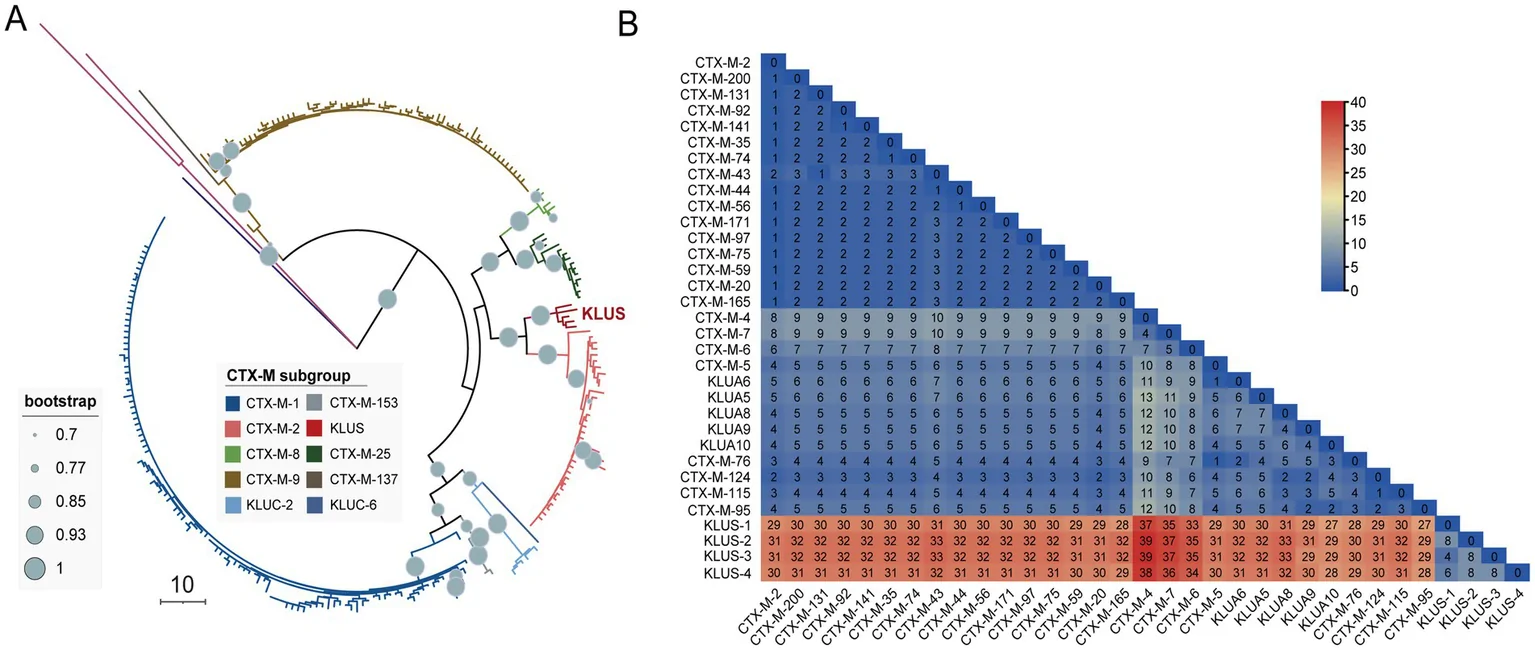
Genetic variation of novel KLUS and known CTX-Ms. **(A)** Phylogenetic analysis of the amino acid sequences of all currently available *bla*_CTX-Ms_ and the genotypes of KLUS using the maximum likelihood method. **(B)** Estimates of evolutionary divergence between KLUS and CTX-Ms sequences. The number represents the amino acid variations across the set of sequences, covering pairwise comparisons with representative members of all established subgroups.

Genetic distance analysis showed mean intra-subgroup genetic distances of 4.6 ± 2.6 for CTX-M-2 and 7 ± 0.5 for KLUS, with no significant difference detected (*p* > 0.05), demonstrating comparable intra-subgroup evolutionary rates. The mean inter-subgroup genetic distance was 10.5 ± 11.3, which was significantly greater than the respective intra-subgroup distances (*p* < 0.05). This finding confirms that the KLUS subgroup represents a novel, independent lineage distinct from CTX-M-2. This marked inter-subgroup genetic divergence (Figure 1B) further validates the taxonomic designation of the KLUS subgroup as an independent evolutionary clade. Moreover, the pairwise distances in Figure 1B show that KLUS differs from representative members of every other established subgroup (the CTX-M-1, CTX-M-8, CTX-M-9, CTX-M-25, KLUA, and KLUC lineages) by 27–39 amino-acid substitutions (Yu et al., 2023), confirming that KLUS satisfies the subgroup criterion family-wide and not only relative to the CTX-M-2 subgroup.

### Recombinant plasmids construction and protein expression

The KLUS subgroup genes were amplified and ligated into pET-28a(+), then transformed into *E. coli* DH5α. Sequencing of PCR-positive clones confirmed 100% sequence identity with the original KLUS genes, with no unintended mutations detected. Double digestion with BamHI/HindIII generated fragments matching the expected sizes. For expression, pET-28a-KLUS was transformed into *E. coli* BL21 (DE3). Initial induction with 1 mM IPTG at 37 °C for 4 h produced target bands on SDS-PAGE (Supplementary Figure S2) consistent with the predicted sizes of the fusion proteins. Optimization identified 1.0 mM IPTG at 30 °C for 5 h as the optimal condition, selected on the basis of the highest soluble target-protein yield as judged by SDS-PAGE densitometry (Supplementary Figures S2B,C).

### Drug resistance phenotype

MIC determinations revealed distinct resistance profiles among KLUS variants (Table 2). All recombinant strains exhibited the high-level resistance to ampicillin and ceftriaxone, followed by cefotaxime, while consistently showing lower resistance to ceftazidime. The rank order of resistance potency among KLUS subgroup variants was determined as follows: KLUS-4 > KLUS-2 > KLUS-3 > KLUS-1. And β-lactamase inhibitors, particularly clavulanic acid and tazobactam, significantly reduced cefotaxime MICs, which confirmed the ESBL activity of KLUS subgroup proteins. Notably, KLUS-4 exhibited a higher level of ceftazidime resistance (MIC = 8 μg/mL, compared with <0.125 μg/mL for KLUS-1–3) and a reduced inhibitor response at the phenotypic level, with a 16-fold reduction in MIC observed for the cefotaxime-clavulanic acid combination (versus far exceeding 16-fold for KLUS-1–3).

**Table 2:** β-lactam susceptibility of *E. coli* BL21 (DE3) strains carrying *bla*_KLUS_ genes.

Drug	MIC (μg/mL)				
	Control	*bla*_KLUS-1_	*bla*_KLUS-2_	*bla*_KLUS-3_	*bla*_KLUS-4_
AMP	4	2048	>2048	>2048	>2048
CRO	0.06	256	>1,024	256	512
CAZ	0.06	<0.125	<0.125	<0.125	8
CTX	0.125	32	128	128	64
CTX + CLA	<0.015	<0.015	<0.015	<0.015	4
CTX + TZB	<0.015	0.03	0.03	0.03	2
CTX + SUL	0.03	2	0.125	0.06	4

Control represents the *E. coli* BL21 carrying the empty pET-28a(+) vector. AMP, Ampicillin; CRO, Ceftriaxone; CAZ, Ceftazidime; CTX, Cefotaxime; CLA, Clavulanic acid; TZB, Tazobactam; SUL, Sulbactam. The concentrations of clavulanic acid, tazobactam, and sulbactam used in the study were 4 μg/mL following the CLSI-standard format. Values are modal MICs from three independent biological replicates and replicate ranges never exceeded one twofold dilution.

### Enzymatic activity and steady-state kinetics

Nitrocefin hydrolysis confirmed functional β-lactamase activity in all KLUS-expressing strains (Supplementary Table S4). Steady-state kinetic analyses (Table 3) revealed subtype-specific substrate preferences among KLUS variants. In KLUS-2/3/4, the highest catalytic efficiency (*k*_cat_/*K*_m_) was observed for ampicillin or penicillin G, consistent with MIC results. In KLUS-1, ceftriaxone instead showed the highest efficiency. All KLUS variants demonstrated the lowest catalytic efficiency for ceftazidime, corroborating their reduced resistance to this cephalosporin. Compared with other subtypes (*K*_m_ = 137–222 μM), KLUS-4 exhibited enhanced affinity for ceftazidime (*K*_m_ = 38 μM), consistent with its elevated ceftazidime MIC. Ceftriaxone and cefotaxime showed subtype-dependent affinities, with KLUS-3 displaying highest ceftriaxone affinity and KLUS-4 lowest cefotaxime affinity. Kinetic data were strongly correlated with MIC profiles, validating that substrate preference serves as a key driver of resistance phenotypes. Some *K*_m_ values exhibited relatively large standard errors (e.g., 45 ± 20 μM for KLUS-1 with penicillin G; 222 ± 106 μM for KLUS-3 with ceftazidime), likely reflecting incomplete saturation for low-turnover substrates due to solubility constraints. The substantially lower *k*_cat_/*K*_m_ for ceftazidime compared to ampicillin may explain why only KLUS-4, with its higher affinity for ceftazidime, conferred elevated ceftazidime resistance.

**Table 3:** Kinetic constants of four purified KLUS enzymes.

Enzyme	Enzyme kinetic parameters	CRO	CTX	CAZ	NCF	AMP	PG
KLUS-1	*K*_m_(μM)	87 ± 23	44 ± 1	137 ± 2	37 ± 3	13 ± 1	45 ± 20
	*k*_cat_(s^−1^)	38 ± 10	6 ± 0.1	1 ± 0.02	15 ± 0.5	2 ± 0.3	13 ± 2.7
	*k*_cat_/*K*_m_(μM^−1^ s^−1^)	0.4368	0.1364	0.0073	0.4054	0.1538	0.2889
KLUS-2	*K*_m_(μM)	63 ± 20	47 ± 13	168 ± 9	58 ± 27	23 ± 10	27 ± 7
	*k*_cat_(s^−1^)	26 ± 0.4	13 ± 0.2	1 ± 0.1	2 ± 0.5	10 ± 2	11 ± 1
	*k*_cat_/*K*_m_(μM^−1^ s^−1^)	0.413	0.277	0.0059	0.0345	0.4348	0.4074
KLUS-3	*K*_m_(μM)	28 ± 6	46 ± 18	222 ± 106	84 ± 26	45 ± 19	25 ± 1
	*k*_cat_(s^−1^)	23 ± 2	8 ± 0.2	3 ± 1	11 ± 2	10 ± 2	28 ± 0.2
	*k*_cat_/*K*_m_(μM^−1^ s^−1^)	0.8214	0.1739	0.0135	0.131	0.222	1.12
KLUS-4	*K*_m_(μM)	91 ± 47	92 ± 38	38 ± 7	50 ± 17	100 ± 1	12 ± 2
	*k*_cat_(s^−1^)	26 ± 8	8 ± 2	1 ± 0.1	14 ± 2	21 ± 0.5	10 ± 3.6
	*k*_cat_/*K*_m_(μM^−1^ s^−1^)	0.2857	0.0869	0.0263	0.28	0.21	0.833

CRO, Ceftriaxone; CTX, Cefotaxime; CAZ, Ceftazidime; NCF, Nitrocefin; AMP, Ampicillin; PG, Penicillin G.

### Structural characteristics and key mutations

The protein structure of the KLUS subgroup consists of 11 α-helices and 5 β-sheets (Figure 2A). The Ω-loop and β3-sheet collectively form the catalytic cavity of the enzyme, which is separated near residue Ambler positions 240 and 170. This structural feature facilitates the entry of the large side chain of cefotaxime molecules, consistent with the structural characteristics of other CTX-M subgroups. The SDN Loop is localized in the core region and adopts a conformation composed of a random coil and a short β-turn. Comparison with other CTX-M family members revealed that the SDN Loop is highly conserved, suggesting its involvement in substrate binding and the stabilization of the catalytic core (Figure 2B).

**Figure 2:**
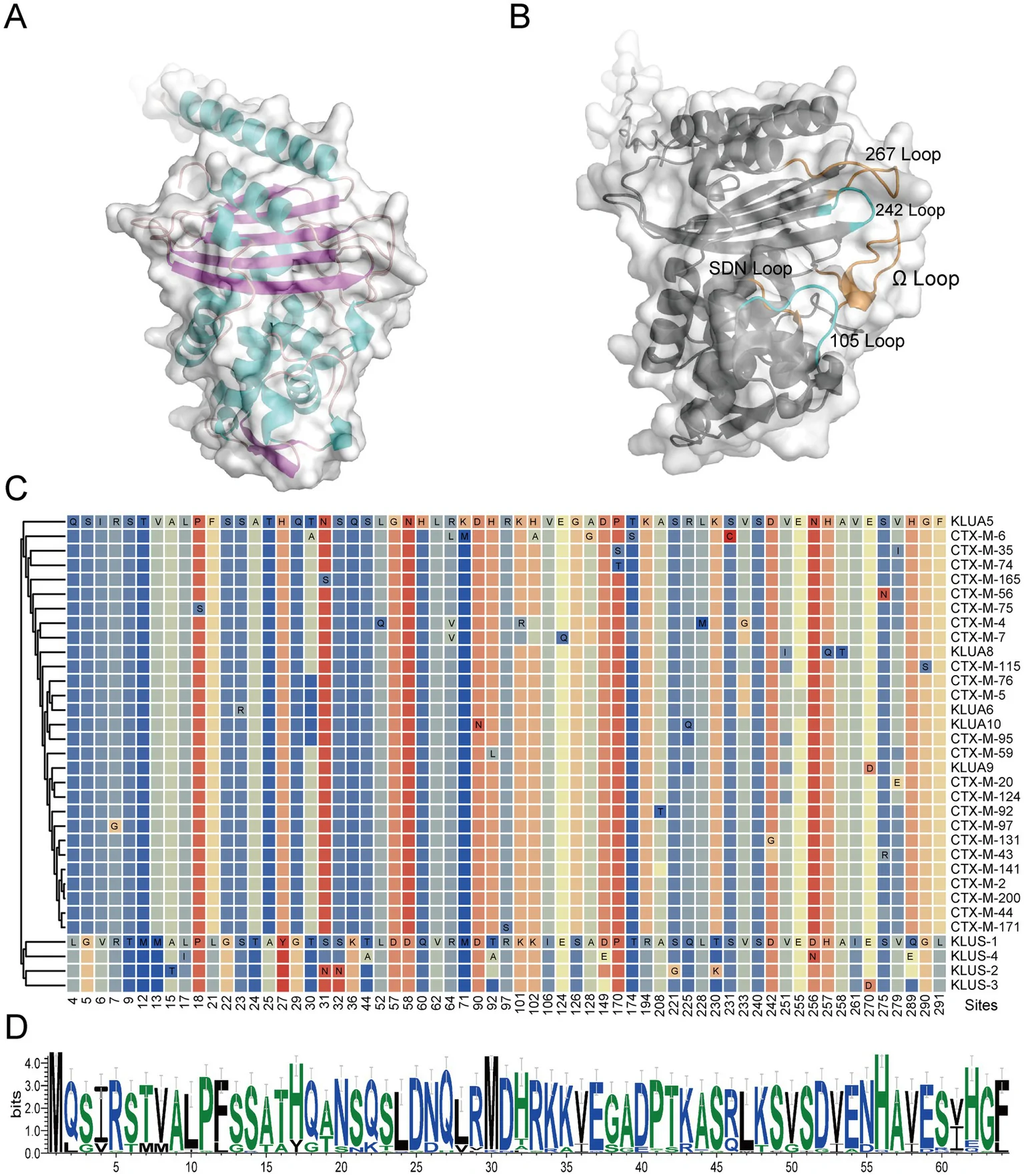
Sequence characteristics and structural analysis of novel KLUS. **(A)** The architecture of KLUS-1. The structure of KLUS-1 (white surface) comprises 11 α-helix (blue) and five β-sheets (pink). **(B)** The conserved loop structures similar to other CTX-M-type β-lactamases. **(C)** Distribution of differential amino acid sites between the KLUS subgroup and the CTX-M-2 subgroup. CTX-M-2-subgroup (pre-protein sequence). Ambler standard numbering differs from the pre-protein alignment positions by 3 residues within the signal-peptide region and by 26 residues in the mature enzyme, owing to the signal-peptide offset in the reference alignment. **(D)** Sequence logo analysis of variable amino acid positions from the alignment of 29 non-redundant CTX-M-2-subgroup sequences together with the four KLUS alleles. Invariant sites were excluded from the analysis. The height of each amino acid residue stack at a given position represents the degree of conservation, with taller stacks indicating higher conservation. The *x*-axis represents the position of amino acid residues in the analyzed sequence segment, and the *y*-axis indicates the information content, reflecting the conservation strength at each position.

Through multiple sequence alignment, 63 differential amino acid sites were identified between CTX-M-2 and KLUS, of which 23 unique substitutions (Ambler numbering: 1, 2, 3, 6, 9, 10, 18, 19, 21, 22, 24, 26, 33, 54, 55, 59, 99, 103, 125, 191, 227, 258, 288) fully distinguish KLUS from CTX-M-2 (Figures 2C,D). Additionally, subgroup-specific mutations were observed at Ambler positions 12, 218, 253. These differential sites are intuitively observable in the multiple sequence alignment diagrams (Figure 2C), providing key evidence for investigating the protein structure and function of the KLUS subgroup, as well as the mechanism underlying subgroup differentiation. The V77I substitution (corresponding to position 106 of the KLUS pre-protein) is located proximal to the Ω-loop region.

## Discussion

Phylogenetic and genetic distance analyses provide robust evidence for the taxonomic independence of the KLUS subgroup. The distinct clustering of KLUS sequences adjacent to the CTX-M-2 clade, accompanied by significant branch separation (bootstrap support >95%), and the substantially greater inter-subgroup compared with intra-subgroup genetic distances fulfill established criteria for defining a new CTX-M subgroup (D'Andrea et al., 2013; Pitout and Laupland, 2008). This evolutionary divergence is further supported by the identification of 23 unique amino acid substitutions that reliably distinguish KLUS from CTX-M-2 variants, which may reflect distinct evolutionary histories of these lineages. The taxonomic independence of KLUS was established in our previous family-wide reclassification of all mature CTX-M sequences, which defined a 10-subgroup scheme based on a ≤ 20-amino-acid-substitution threshold (>93.13% identity) validated against phylogenetic clustering (Yu et al., 2023). The present analysis included representative members of all established subgroups (Figure 1A; Supplementary Figure S1) and revealed inter-subgroup distances of 27–39 amino acid substitutions between KLUS and all other subgroups (Figure 1B). These findings are fully consistent with that framework and extend it from taxonomy to the functional and structural consequences of KLUS divergence. The successful heterologous expression of KLUS enzymes using the pET-28a(+)/*E. coli* BL21 (DE3) system confirms the structural integrity of these β-lactamases and provides a robust platform for functional characterization. The optimized expression conditions yielded soluble recombinant proteins with molecular weights consistent with in silico predictions, thereby enabling detailed enzymatic analyses. This experimental system represents a valuable resource for future structure–function studies of these novel enzymes.

The resistance phenotypes exhibited by KLUS-expressing strains are consistent with characteristic ESBL profiles but reveal distinctive substrate preferences that may have clinical implications. The consistent hierarchy of resistance (ampicillin = ceftriaxone > cefotaxime > ceftazidime) and significant susceptibility to β-lactamase inhibitors (particularly clavulanic acid) confirm KLUS as an authentic ESBL. However, the unique phenotype of KLUS-4, characterized by elevated ceftazidime resistance and a reduced inhibitor response at the phenotypic level, warrants particular attention. Whether this phenotype reflects adaptation to cephalosporin pressure cannot be determined from the present data. In any case, it flags KLUS-4 as a variant meriting surveillance (Djamdjian et al., 2011; Yahav et al., 2020). As these findings derive from recombinant laboratory strains, phenotypes in native hosts or clinical isolates may differ, and direct clinical extrapolation is premature.

The genomic context of the *bla*_KLUS_ alleles provides further perspective on the threat level of this subgroup. In our previous genomic study (Yu et al., 2023), all four alleles were found to be chromosomally located in their *Kluyvera* hosts. Screening of 10 kb flanking sequences against the ISfinder database identified no ISEcp1, ISCR1, transposons, integrons or plasmid replicons, and the loci sit within conserved chromosomal neighborhoods. This architecture is consistent with *Kluyvera* chromosomes acting as the progenitor reservoir of CTX-M/KLU-type enzymes (Rodríguez et al., 2021) and indicates that the KLUS alleles characterized here are not currently associated with known mobilization elements, representing a reservoir of potential future mobilization rather than an actively disseminating threat. More broadly, the emergence and diversification of β-lactamase subgroups are embedded in the complex dynamics of the resistome and mobilome, in which mobile genetic elements, integrons, and accessory-genome plasticity drive the capture and rapid dissemination of chromosomal β-lactamase progenitors into pathogenic Enterobacterales (Biswas and Anbarasu, 2026). Continued genomic surveillance of *Kluyvera* reservoirs is therefore warranted.

Enzymatic kinetic analyses provide mechanistic explanations for the observed resistance phenotypes, establishing substrate affinity (*K*_m_) and catalytic efficiency (*k*_cat_/*K*_m_) as key determinants of antimicrobial susceptibility. The correlation between high catalytic efficiency for ampicillin and penicillin G and elevated MICs for these agents confirms the functional relevance of these kinetic parameters. Notably, the enhanced ceftazidime affinity of KLUS-4 compared with other KLUS variants directly correlates with its higher ceftazidime MIC, illustrating how subtle changes in substrate binding can translate to clinically significant resistance phenotypes. Nevertheless, without Ki or IC50 determinations, the mechanistic basis for the altered inhibitor response of KLUS-4 cannot be established. Moreover kinetic characterization was limited to six substrates, precluding full mechanistic interpretation of the inhibitor susceptibility patterns. The consistently lower catalytic efficiency for ceftazidime across all KLUS variants provides a molecular basis for their reduced resistance to this cephalosporin. However, because active-site titration was not performed, the reported *k*_cat_ values are apparent estimates referenced to total enzyme concentration. And the *K*_m_ estimates with large standard errors (KLUS-3/ceftazidime; KLUS-1/penicillin G) are apparent, lower-bound estimates, as substrate solubility precluded full saturation for these low-turnover pairs. Importantly, the *k*_cat_/*K*_m_ ratios that underpin our conclusions are determined by the linear, sub-saturating region of the curves and are minimally affected by this uncertainty, and the kinetic ranking is independently corroborated by the MIC data.

Beyond the kinetic level, the evolution of CTX-Ms has been shaped by accumulated polymorphic substitutions. To date, at least 136 polymorphic sites have been identified in CTX-Ms. Variations at some of these sites can expand the hydrolytic spectrum of β-lactamases or regulate and reverse changes in substrate spectra caused by other modifications, exerting either synergistic or antagonistic effects. The selection of mutations depends on the genetic background, and epistatic interactions between mutations also provide new insights into the evolutionary history of CTX-Ms. Under laboratory conditions, strains carrying the S130G, K234R, and S237A mutations lose their hydrolytic activity against cefotaxime and ceftazidime (Aumeran et al., 2003; Ripoll et al., 2011). However, the L169S mutation, which is restored following the selection of S130G, slightly enhances the hydrolytic efficiency of strain against cefotaxime and ceftazidime (Novais et al., 2008; Ripoll et al., 2011). Mutations at position 169 increase the enzymatic hydrolytic activity against ceftazidime, and this improvement is more pronounced when the S130G mutation is also present.

Structural analyses reveal both conserved features and unique adaptations within KLUS enzymes. The preservation of 11 α-helices and 5 β-sheets, along with the conserved architecture of the Ω-loop and β3-sheet forming the catalytic cavity, reflects the structural constraints required for β-lactam hydrolysis. The conserved SDN Loop conformation aligns with its proposed role in substrate binding and catalytic core stabilization across the CTX-M family. The unique amino acid substitutions identified in KLUS, including those within the predicted signal peptide and at Ambler positions 77 and 262 of the mature enzyme, are localized peripherally to the active center, suggesting they may influence protein stability or substrate access rather than directly participating in catalysis, although this remains to be tested experimentally. The V77I substitution (pre-protein position 106) is located within an α-helical cluster, and its hydrophobic interactions with adjacent residues (L81, L127, L138, W210) suggest that it could modulate the structure of the 105 loop and thereby substrate specificity. This hypothesis requires experimental validation. The 63 amino acid differences between the KLUS and CTX-M-2 subgroups, including 23 KLUS-specific substitutions, provide valuable insights into the evolutionary dynamics of CTX-M diversification. Whether the 23 KLUS-specific substitutions reflect adaptation to distinct selective pressures, neutral drift accumulated in different *Kluyvera* hosts, or a combination of both cannot be determined from the present data and requires experimental investigation of protein stability and fitness effects. The subgroup-specific substitutions (Ambler positions 12, 218, and 253) merit further investigation as potential determinants of host specificity or substrate range expansion. Because the structural analyses are based on static homology models, no conclusions about loop flexibility or substrate-entry dynamics are drawn. Molecular dynamics simulations and experimental structures will be required to address these aspects. The functional significance of the identified amino acid substitutions requires confirmation through site-directed mutagenesis studies, particularly for the V77I substitution and the T44A variation in KLUS-4, which may contribute to its unique ceftazidime resistance profile. Crystallographic analyses would provide definitive insights into how these substitutions affect protein structure and substrate interactions. Additionally, the clinical relevance of KLUS enzymes requires evaluation through expanded epidemiological surveys to determine their prevalence and association with treatment failures.

## Data Availability

The datasets presented in this study can be found in online repositories. The names of the repository/repositories and accession number(s) can be found in the article/Supplementary material.

## Footnotes

1
http://bldb.eu/

2
http://swissmodel.expasy.org
