# Supplementary material for: Genetic, biochemical, and structural characterization of a novel KLUS subgroup of CTX-M-type extended-spectrum β-lactamases reveals an evolutionarily distinct lineage with unique functional properties
Source: Front Microbiol. 2026 Oct 30;17:1997337. doi: 10.3389/fmicb.2026.1997337 (PMC13759820; doi:10.3389/fmicb.2026.1997337)
Supplement: Supplementary file 1 [file Supplementary_file_1.docx]

Genetic, Biochemical, and Structural Characterization of a Novel KLUS Subgroup of CTX-M-Type Extended-Spectrum β-Lactamases Reveals an Evolutionarily Distinct Lineage with Unique Functional Properties

Zhenzhou Huang^1,2#^, Jun Li^1,2#^, Xiao Liu^3^, Lingyi Zeng^1,2^, Zhibei Zheng^1,2^, Hua Yu^1,2^, Duochun Wang^3*^, Keyi Yu^4,5,6*^, Junfang Chen^1,2*^

^1^Hangzhou Center for Disease Control and Prevention (Hangzhou Health Supervision Institution), Hangzhou, Zhejiang Province, China

^2^Zhejiang Key Laboratory of Multi-Omics in Infection and Immunity, Hangzhou, Zhejiang Province, China

^3^National Key Laboratory of Intelligent Tracking and Forecasting for Infectious Diseases, National Institute for Communicable Disease Control and Prevention, Chinese Center for Disease Control and Prevention & Chinese Academy of Preventive Medicine, Beijing, China

^4^Department of Microbiology, Zhejiang Provincial Center for Disease Control and Prevention, Hangzhou, Zhejiang Province, China

^5^NHC Specialty Laboratory of Food Safety Risk Assessment and Standard Development, Zhejiang Provincial Center for Disease Control and Prevention, Hangzhou, China

^6^Zhejiang Key Laboratory of Public Health Detection and Pathogenesis Research, Zhejiang Provincial Center for Disease Control and Prevention, Hangzhou, China.

*Correspondence Authors:

Junfang Chen, E-mail: hzjkcjf@hzcdc.com.cn; Address: 568 Mingshi Road, Shangcheng District, Hangzhou, Zhejiang Province, 310021, P.R. China.

Duochun Wang, E-mail: wangduochun@icdc.cn; Address: 155 Changbai Road, Changping District, Beijing, 102206, P.R. China.

Keyi Yu, E-mail: kyyu@cdc.zj.cn; Address: 3399 Binsheng Road, Binjiang District, Hangzhou, Zhejiang Province, 310051, P.R. China.

^#^ These authors contributed equally to this work

**Supplementary Table**

| Table S1. The detail information of ***Kluyvera*** strains used in this study | | | | | | |
| --- | --- | --- | --- | --- | --- | --- |
| **No.** | **Strain** | **Region** | **Isolation Time** | **Isolation Source** | **Species** | **Accession Number** |
| 1 | 090646 | Chengdu, China | 2019 | hospital sink | *Kluyvera sichuanensis* | GCA_014218705.1 |
| 2 | CHPC 1.251 | Beijing, China | 2007 | feces | *Kluyvera excreta* | GCA_036759945.1 |
| 3 | CHPC 1.254 | Beijing, China | 2007 | feces | *Kluyvera sichuanensis* | GCA_036711835.1 |
| 4 | CHPC 1.2972 | Beijing, China | 2016 | egg surface | *Kluyvera chilikensis* | GCA_036759925.1 |

| **Table S2. Primer sequences of *bla*_CTX-Ms_ and length of amplified fragments** | | | |
| --- | --- | --- | --- |
| **Target gene** | **Primer sequence（5’-3’）** | **Product length** | **Annealing temperature** |
| KLUS 1 | F：CGGGATCCATGATGACCCTGGGCGTTCG | 876 bp | 54℃ |
|  | R：CCCAAGCTTCTAAAGCCCTTGGGTAACG |  |  |
| KLUS 2 | F：CGGGATCCATGATGACCCTGGGCGTTCG | 915 bp | 53℃ |
|  | R：CCCAAGCTTCTAAAGCCCTTCGGTAACG |  |  |
| KLUS 3 | F：CGGGATCCATGATGACCCTGGGCGTTCG | 876 bp | 57℃ |
|  | R：CCCAAGCTTCTAAAGCCCTTGGGTAACG |  |  |
| KLUS 4 | F：CGGGATCCATGATGACCCTGGGCGTTCG | 876 bp | 54℃ |
|  | R：CCCAAGCTTCTAAAGCCCTTGGGTAACG |  |  |
| Note：The target genes KLUS1-4 represent *bla*_KLUS-1_ (isolated from strain 090646), *bla*_KLUS-2_ (isolated from CHPC 1.251), *bla*_KLUS-3_ (isolated from CHPC 1.254), *bla*_KLUS-4_ (isolated from CHPC 1.2972). | | | |

| **Table S3. Purification summary of the four recombinant KLUS β-lactamases** | | | | | | |
| --- | --- | --- | --- | --- | --- | --- |
| **Enzyme** | **Purification step** | **Total protein (mg)** | **Total activity (U)** | **Specific activity (U/mg)** | **Purification (fold)** | **Yield (%)** |
| KLUS-1 | Crude lysate | 124.7 | 204.5 | 1.64 | 1.00 | 100.0 |
|  | Flow-through | 89.8 | 5.3 | 0.06 | 0.04 | 2.6 |
|  | Wash | 11.9 | 0.9 | 0.08 | 0.05 | 0.4 |
|  | Eluate | 4.0 | 117.6 | 29.40 | 17.93 | 57.5 |
| KLUS-2 | Crude lysate | 96.3 | 21.2 | 0.22 | 1.00 | 100.0 |
|  | Flow-through | 76.1 | 1.7 | 0.02 | 0.10 | 8.0 |
|  | Wash | 8.7 | 0.2 | 0.02 | 0.10 | 0.9 |
|  | Eluate | 2.7 | 10.1 | 3.74 | 16.99 | 47.6 |
| KLUS-3 | Crude lysate | 109.8 | 132.9 | 1.21 | 1.00 | 100.0 |
|  | Flow-through | 78.0 | 4.1 | 0.05 | 0.04 | 3.1 |
|  | Wash | 10.4 | 0.8 | 0.08 | 0.06 | 0.6 |
|  | Eluate | 3.3 | 68.2 | 20.67 | 17.07 | 51.3 |
| KLUS-4 | Crude lysate | 117.2 | 164.1 | 1.40 | 1.00 | 100.0 |
|  | Flow-through | 80.9 | 4.6 | 0.06 | 0.04 | 2.8 |
|  | Wash | 14.1 | 1.8 | 0.13 | 0.09 | 1.1 |
|  | Eluate | 3.3 | 86.3 | 26.15 | 18.68 | 52.6 |
| Note: One unit (U) of activity is defined as 1 μmol of nitrocefin hydrolyzed per minute at 25 °C in 50 mM phosphate buffer (pH 7.0), monitored at 482 nm (Δε = 15,000 M⁻¹cm⁻¹). Protein was quantified by the BCA method. | | | | | | |

| **Table S4. Difference in OD468 of pET-28a(+)/BL21 and recombinants** | | | | | | |
| --- | --- | --- | --- | --- | --- | --- |
|  | **A** | **B** | **C** | **D** | **E** | **F** |
| **OD_468_** | 1.749 | 1.925 | 3.767 | 3.526 | 3.862 | 3.774 |
| **Fold increase** | - | - | 2.0506 | 1.9194 | 2.1023 | 2.0544 |

**Supplementary Figure**

**Fig. S1. Rectangular cladogram of the phylogenetic tree shown in Fig. 1A.** Fully resolved rectangular representation with readable strain identifiers and bootstrap values at all nodes. The basal nodes defining the KLUS clade are indicated.

**Fig. S2. Protein Expression of Novel KLUS.** (A) SDS-PAGE results of KLUS. The band indicated by the arrow corresponds to the specific band of the fusion protein, which is consistent with the expected size. B: SDS-PAGE results for KLUS with different concentrations of IPTG. 1: Induction without IPTG addition; 2-4: Results of induction with 0.5 mM, 1 mM, and 2 mM IPTG, respectively. C: SDS-PAGE results for KLUS at different temperatures. 1: Induction at 16°C for 18 hours; 2: Induction at 30°C for 5 hours; 3: Induction at 37°C for 4 hours. M: Protein Marker, used as a reference for protein molecular weight standards.
